# Life history of the most complete fossil primate skeleton: exploring growth models for *Darwinius*

**DOI:** 10.1098/rsos.150340

**Published:** 2015-09-09

**Authors:** Sergi López-Torres, Michael A. Schillaci, Mary T. Silcox

**Affiliations:** Department of Anthropology, University of Toronto Scarborough, 1265 Military Trail, Toronto, Ontario, Canada M1C 1A4

**Keywords:** Adapoidea, Strepsirrhini, Haplorhini, Anthropoidea, Eocene

## Abstract

*Darwinius* is an adapoid primate from the Eocene of Germany, and its only known specimen represents the most complete fossil primate ever found. Its describers hypothesized a close relationship to Anthropoidea, and using a *Saimiri* model estimated its age at death. This study reconstructs the ancestral permanent dental eruption sequences for basal Euprimates, Haplorhini, Anthropoidea, and stem and crown Strepsirrhini. The results show that the ancestral sequences for the basal euprimate, haplorhine and stem strepsirrhine are identical, and similar to that of *Darwinius*. However, *Darwinius* differs from anthropoids by exhibiting early development of the lower third molars relative to the lower third and fourth premolars. The eruption of the lower second premolar marks the point of interruption of the sequence in *Darwinius*. The anthropoid *Saimiri* as a model is therefore problematic because it exhibits a delayed eruption of P_2_. Here, an alternative strepsirrhine model based on *Eulemur* and *Varecia* is presented. Our proposed model shows an older age at death than previously suggested (1.05–1.14 years), while the range for adult weight is entirely below the range proposed previously. This alternative model is more consistent with hypotheses supporting a stronger relationship between adapoids and strepsirrhines.

## 1. Background

Adapoids were medium-sized, arboreal euprimates, widespread throughout portions of Europe, Asia, Africa and North America from the Early Eocene to the Late Miocene. Adapoids were a very diverse group, comprising six families and more than 100 species [1–3]. Despite receiving considerable attention in the literature, the evolutionary relationships of adapoids to modern strepsirrhines or haplorhines remain unclear. Currently, two opposing hypotheses predominate: (i) the Adapoid–Anthropoid hypothesis [4–17] and (ii) the Adapoid–Strepsirrhine hypothesis [18–25].

The Adapoid–Strepsirrhine hypothesis has been more broadly accepted for the past two decades, based on the fact that it is recovered in all broadly sampled, recent phylogenetic analyses [22,26–29]. However, the debate surrounding the phylogenetic position of adapoids was thrust back into prominence with the description of the most complete fossil primate skeleton, the caenopithecid adapoid *Darwinius masillae*. Franzen *et al*. [17] proposed a stronger relationship between *Darwinius* and haplorhines, and Gingerich *et al*. [30] later united it specifically with anthropoids. Since then, the biology and the evolutionary relationships of *Darwinius* and adapoids to modern primates have been discussed extensively in the literature (e.g. [23,24,31–36]). An area that has received much less treatment than the question of *Darwinius*' phylogenetic position is the age model used [17] to reconstruct its body mass and age at death. This paper considers the appropriateness of anthropoids generally, and *Saimiri* specifically, with respect to choosing an appropriate model for *Darwninius*' development, and also assesses the relevance of growth data to the phylogenetic debates.

### 1.1 Relevance of dental eruption sequences in primate life history

Life-history analysis assesses the chronology of development and reproduction throughout a species' lifetime, from conception to death. The pattern and timing of tooth emergence known for extant primates can be used to broadly infer the life history of fossil groups [37–39]. This is particularly useful for fossil taxa, in which the dentition is the primary and most reliable gauge of ontogeny [39]. The relevance of the dentition as a source of information to explain life history relies on the fact that it is not strongly influenced by environment [40]. There is also a relationship between the sequence of dental eruption and the overall pace of growth, maturation and other aspects of primate life history [41]. For example, M_1_ is the first permanent tooth to erupt in primates, and the timing of its emergence is highly correlated with adult brain weight, body weight and probably reflects infant precociality [40,42]. In order to use data from dental eruption sequences in modern taxa to form inferences about a fossil, it is necessary to define the point of interruption of the sequence. Defining this point allows for modern growth trajectory data to be used to determine not only age at death but also other variables such as projected adult weight (e.g. [17]).

### 1.2 Dental eruption and life history of *Darwinius*

The *Darwinius* specimen is split into two parts: PMO 214.214 and its counterpart WDC-MG-210 [17].^1^ The specimens, altogether nicknamed ‘Ida’, come from the site of Messel (near Darmstadt, Germany; 47.5 Ma [43]). The individual was a weaned and independently feeding juvenile, with an erupted M_1_ [17]. Based on the absence of a baculum, the specimen has been interpreted as female [17], although it is possible that this element was lost (e.g. along with the left lower limb below the knee) or had not fully developed at the time of the animal's death.

**Figure 1.**
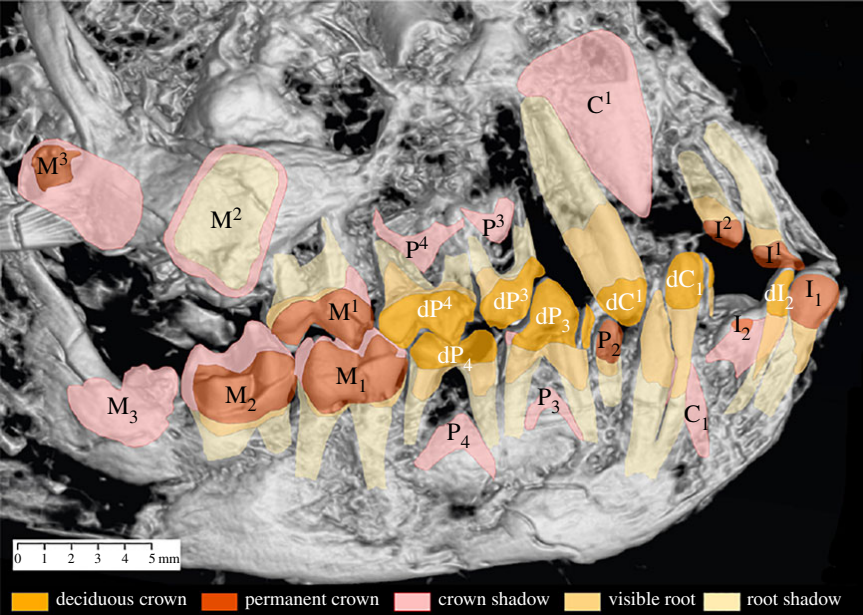
Radiograph of the right side of the skull of *Darwinius masillae* showing the deciduous (indicated with a ‘d’) and permanent teeth. Adapted from Franzen *et al*. [17], fig. 5.

The preserved dentition (figure 1) allows for several inferences about the permanent dental eruption sequence. We follow Gingerich & Smith's [44] inference that P_2_ is an adult tooth, and Franzen *et al*. [17] in considering it to be the last tooth to have emerged before death. It is known that variability in the premolar eruption sequence is common in some New World monkeys, such as in *Callicebus*, *Alouatta*, *Saimiri*, *Callithrix*, *Mico*, *Saguinus* and *Cebus* [45–47]. However, there is no intraspecific variability reported in the presence of P_2_ in adapoids [48,49], and no reported variation in eruption sequences [44]. Therefore, *a priori*, there is no empirical basis for believing the eruption of P_2_ in *Darwinius* is variable, or that P_2_ erupted unusually early or late in ‘Ida’. We are additionally assuming that dP_2_ is replaced by P_2_ in ‘Ida’, as it is in modern primates that retain this tooth. It is possible that this is an incorrect assumption, and that P_2_ emerged without having a deciduous precursor, in a manner similar to P_1_ in many living mammals [50]. However, loss of P_1_ replacement is inferred to be a very ancient feature in mammalian evolution, based on its absence in even some non-eutherians (e.g. *Didelphis* [51]), whereas in all adapoids for which there are adequate data, P_2_ is known to be replaced [18,44]. As such, arguing for a lack of a deciduous precursor for P_2_ would require the assumption that this particular lineage developed a peculiar homoplasy, not observed in living primates. Although this is of course possible, in the absence of any data it seems more parsimonious to assume that *Darwinius* was a typical adapoid and replaced its P_2_. It is worth noting that in applying a *Saimiri* model, or indeed a model based on any living primate (e.g. [17,44]), this same assumption is being made.

Following from this inference, the teeth of *Darwinius* can be divided in two sets: (M_1_ M_2_ I_1_ P_2_), which are adult and erupted, and (I_2_ M_3_ C P_4_ P_3_), which are in various stages of development and have yet to erupt, with the point of interruption in the sequence occurring after the eruption of P_2_ [17]. Using the dental eruption sequence of the New World anthropoid *Saimiri* as a model, Franzen *et al*. [17] estimated an age at death between 9 and 10 months based on the interruption of the dental eruption sequence, and a projected adult weight between 650 and 900 g. For the unerupted teeth, it is also possible to make certain inferences about their likely place in the sequence based on their degree of development. For example, the crown of M_3_ is completely formed and the tooth is in process of erupting. It was probably only covered by soft tissue, although it still lacks mineralized roots [17]. Therefore, this would place the timing of eruption of M_3_ early in the sequence.

The goals of the present study are to reassess the age estimate for *Darwinius* by using ancestral reconstruction of dental eruption sequences, and to further explore the life history of *Darwinius*. Specifically, we assess the validity of using the anthropoid (*Saimiri*) model for inferring the age and life history of *Darwinius*.

## 2. Material and methods

Ancestral state reconstruction has been embraced by both systematists and evolutionary biologists for many years [52] as a way of making inferences about the pattern of evolution of particular traits or character complexes. Indeed its implementation can be traced back almost 30 years to the work of Felsenstein [53]. Since then, this method has been applied extensively by many authors in the field of palaeontology (e.g. [54–64]). For the ancestral state reconstruction analysis in this study, dental eruption sequences for 97 fossil and extant taxa were used, including Primates, Scandentia and Soricomorpha (table 1). A matrix of 14 characters related to eruption sequences was created (table 2). The 14 characters used in our matrix constitute the minimum number of informative characters needed to allow the reconstruction of eruption sequences. These characters code for presence or absence of certain teeth, and relative timing of eruption of teeth in different positions. By reconstructing the ancestral states for every character, it is possible, from the information they provide, to infer the order of dental eruption at any particular node on the tree. Although not all of the characters are independent, because of the way they are defined, it is impossible for them to generate conflicting reconstructions. For example, in electronic supplementary material, table S2, the ancestral reconstruction for the euprimate node for character 6 ‘Eruption of I_1_ relative to the earliest premolar’ recovers both states (i.e. I_1_ erupting before or after the earliest premolar), instead of one ancestral state. However, because all incisors erupt unequivocally before M_2_ (character 8, state 3) and all premolars erupt unequivocally after M_2_ (character 12, state 0), the ancestral state for character 6 resolves as I_1_ erupting before the earliest premolar.

**Table 1.** List of lower permanent dental eruption sequences for 97 taxa. Taxa marked with an asterisk (*) had published information on only upper dentition. The time of eruption between lower and upper dentition differs, but the sequence of eruption is usually the same for both dentitions. Parentheses () group teeth that in a fossil are either all emerged or all have not emerged yet. Square brackets [ ] surround teeth when actual sequence has not yet been resolved. Simultaneous eruptions are indicated with teeth united by hyphens, i.e. toothcombs.

taxon	permanent dental eruption sequence
***Darwinius masillae***[17]	**(M**_**1**_**M**_**2**_**I**_**1**_**P**_**2**_**)(I**_**2**_**C M**_**3**_**P**_**4**_**P**_**3**_**)**^a^
*Dymecodon pilirostris*[65]	M_1_ M_2_ M_3_ [P_2_ P_3_ P_4_] I_1_ C P_1_
*Tupaia glis*[65]	M_1_ M_2_ M_3_ P_2_ I_3_ P_4_ [I_1_ C] P_3_ I_2_
*Acidomomys hebeticus*[66]	M_1_ M_2_ P_3_ (I_1_ M_3_) (I_2_ P_4_)
Plesiadapidae [67]	[M_1_ M_2_ P_2_] [M_3_ P_3_ P_4_]
*Microcebus murinus*[68]	M_1_ M_2_ I_1_-I_2_-C P_2_ M_3_ P_4_ P_3_
*Mirza coquereli*[68]	M_1_ I_1_-I_2_-C M_2_ P_2_ M_3_ P_4_ P_3_
*Cheirogaleus major*[68]	[M_1_ I_1_-I_2_-C M_2_] P_2_ M_3_ P_4_ P_3_
*Cheirogaleus medius*[68]	[M_1_ I_1_-I_2_-C M_2_] P_2_ M_3_ P_4_ P_3_
*Allocebus trichotis*[68]	[M_1_ I_1_-I_2_-C M_2_] P_2_ M_3_ P_4_ P_3_
*Megaladapis edwardsi*[69]	M_1_ I_1_-I_2_-C M_2_ P M_3_ PP
*Lepilemur mustelinus*[68]	M_1_ M_2_ I_1_-I_2_-C P_4_ M_3_ P_3_ P_2_
*Archaeolemur majori*[68,69]	M_1_ M_2_ I_1_-I_2_ M_3_ P_4_ P_3_ P_2_
*Archaeolemur edwardsi*[69]	M_1_ M_2_ PP M_3_ IIP
*Hadropithecus stenognathus*[68,69]	M_1_ M_2_ I_1_-I_2_ M_3_ P_4_ P_3_ P_2_
*Avahi laniger*[68,69]	M_1_ I_1_-I_2_ P_4_ P_3_ M_2_ M_3_
*Propithecus verreauxi*[68,69]	M_1_ I_1_-I_2_ M_2_ P_4_ M_3_ P_3_
*Propithecus diadema*[68,69]	M_1_ I_1_-I_2_ M_2_ P_4_ P_3_ M_3_
*Hapalemur griseus*[68]	M_1_ I_1_-I_2_-C M_2_ P_4_ M_3_ P_3_ P_2_
*Lemur catta*[42,70]	M_1_ M_2_ I_1_-I_2_-C P_4_ P_3_ P_2_ M_3_
*Eulemur mongoz*[71]	M_1_ I_1_-I_2_-C M_2_ M_3_ P_2_ P_4_ P_3_
*Eulemur rufus*[42,70]	M_1_ I_1_-I_2_-C M_2_ P_2_ M_3_ P_4_ P_3_
*Eulemur macaco*[71,72]	M_1_ I_1_-I_2_-C M_2_ P_2_ M_3_ P_4_ P_3_
*Varecia* sp. [42,73]	M_1_ I_1_-I_2_-C M_2_ P_2_ M_3_ P_4_ P_3_
*Otolemur crassicaudatus*[74]	M_1_ I_1_-I_2_-C M_2_ M_3_ P_2_ P_4_ P_3_
*Sciurocheirus alleni*[74]	M_1_ I_1_-I_2_-C M_2_ P_2_ M_3_ P_4_ P_3_
*Galago senegalensis*[74]	M_1_ I_1_-I_2_-C M_2_ P_2_ M_3_ P_4_ P_3_
*Galago gallarum*[74]	M_1_ I_1_-I_2_-C M_2_ M_3_ P_2_ P_4_ P_3_
*Galago moholi*[74]	M_1_ I_1_-I_2_-C P_2_ M_2_ M_3_ P_4_ P_3_
*Galagoides demidovii*[74]	M_1_ M_2_ I_1_-I_2_-C M_3_ P_2_ P_4_ P_3_
*Loris tardigradus*[74]	I_1_-I_2_-C M_1_ M_2_ P_2_ M_3_ P_4_ P_3_
*Nycticebus javanicus*[74]	I_1_-I_2_-C M_1_ M_2_ P_2_ M_3_ P_4_ P_3_
*Nycticebus coucang*[74]	I_1_-I_2_-C-M_1_ M_2_ P_2_ M_3_ P_4_ P_3_
*Perodicticus potto*[74]	I_1_-I_2_-C M_1_ P_2_ M_2_ M_3_ P_4_ P_3_
*Notharctus tenebrosus*[18,44]	M_1_ P_1_ M_2_ M_3_ P_2_ P_4_ P_3_
*Adapis parisiensis*[2,44,75]	M_1_ P_1_ M_2_ M_3_ P_4_ P_3_ P_2_
*Sivaladapis nagrii*[1]	M_1_ I_1_ P_2_ (C P_4_) P_3_
Tarsiidae [76]	[M_1_ P_2_] I_1_ M_2_ M_3_-C P_4_ P_3_
Homunculus patagonicus* [77]	[M_1_ I_1_ I_2_] M_2_ P_2_ P_4_ P_3_ M_3_ C
*Saguinus fuscicollis*[78]	M_1_ I_1_ I_2_ M_2_ P_4_ P_2_ P_3_ C
*Saguinus oedipus*[46]	M_1_ I_1_ [I_2_ M_2_] [P_4_ P_2_] P_3_ C
*Saguinus midas*[46]	M_1_ I_1_ I_2_ M_2_ P_4_ P_2_ P_3_ C
*Saguinus mystax*[46]	M_1_ I_1_ I_2_ M_2_ [P_4_ P_2_] P_3_
*Saguinus bicolor*[46]	M_1_ I_1_ [I_2_ M_2_] P_4_ P_2_ P_3_
*Leontopithecus* sp. [79]	M_1_ I_1_ I_2_ M_2_ P_2_ P_3_ P_4_ C
*Callimico goeldii*[79]	M_1_ [M_2_ I_1_ I_2_] [P_4_ P_2_ P_3_ M_3_ C]
*Cebuella pygmaea*[79]	M_1_ [M_2_ I_1_ I_2_] [P_4_ P_2_ P_3_] C
*Callithrix jacchus*[80,81]	M_1_ M_2_ I_1_ [I_2_ P_4_] P_3_ [P_2_ C]
*Mico argentatus*[46,81]	M_1_ [M_2_ I_1_] [I_2_ P_4_] P_3_ P_2_ C
*Mico humeralifer*[46,81]	M_1_ M_2_ [I_1_ I_2_] P_2_ P_4_ P_3_ C
*Aotus trivirgatus*[65]	M_1_ M_2_ I_1_ M_3_ I_2_ P_4_ P_3_ P_2_ C
*Cebus capucinus*[79]	M_1_ I_1_ I_2_ M_2_ P_4_ P_3_ P_2_ [C M_3_]
*Cebus albifrons*[79]	M_1_ I_1_ I_2_ M_2_ P_2_ P_3_ P_4_? [C M_3_]
*Sapajus apella*[79]	M_1_ I_1_ I_2_ M_2_ P_4_ P_2_ P_3_ C M_3_
*Saimiri sciureus*[70]	M_1_ M_2_ I_1_ I_2_ M_3_ P_4_ P_2_ P_3_
*Alouatta* sp. [79]	M_1_ I_1_ I_2_ M_2_ P_2_ P_3_ [P_4_ M_3_] C
Stirtonia victoriae* [47]	(M_1_ I_1_ I_2_) M_2_ (P_2_ P_4_ P_3_ M_3_ C)
*Lagothrix* sp. [79]	M_1_ I_1_ I_2_ M_2_ P_2_ P_3_? P_4_? M_3_ C
*Ateles* sp. [79]	M_1_ I_1_ I_2_ P_2_ M_2_ P_4_ P_3_ C M_3_
*Brachyteles* sp. [79]	M_1_ I_1_ I_2_ M_2_ P_2_ P_3_ P_4_? C M_3_
*Chiropotes* sp. [79]	M_1_ I_1_ I_2_ M_2_ P_2_ P_3_ P_4_? C M_3_
*Cacajao* sp. [79]	M_1_ M_2_ I_1_ I_2_ P_2_? P_3_? P_4_? M_3_ C
*Pithecia* sp. [79]	M_1_ M_2_ I_1_ I_2_ M_3_ P_4_ P_2_ P_3_ C
*Callicebus* sp. [79]	M_1_ I_1_ I_2_ M_2_ P_2_ P_4_ P_3_ M_3_ C
*Apidium phiomense*[66]	M_1_ M_2_ P_2_ P_4_ (P_3_ M_3_) C
*Parapithecus grangeri*[72]	M_1_ M_2_ P_2_ P_4_ (P_3_ M_3_) C
*Chlorocebus pygerythrus*[65]	M_1_ I_1_ I_2_ M_2_ P_4_ P_3_ C M_3_
Cercopithecus ascanius* [82]	M_1_ I_1_ I_2_ M_2_ [P_3_ P_4_] M_3_ C
*Macaca nemestrina*[83]	M_1_ I_1_ I_2_ M_2_ P_4_ P_3_ C M_3_
*Macaca mulatta*[83]	M_1_ I_1_ I_2_ M_2_ P_3_ P_4_ C M_3_
*Macaca fascicularis*[84]	M_1_ II M_2_ PP M_3_
*Paradolichopithecus arvernensis*[74]	M_1_ I_1_ I_2_ M_2_ P_3_ P_4_ C M_3_
*Papio anubis*[83]	M_1_ I_1_ I_2_ M_2_ C [P_4_ P_3_] M_3_
*Papio cynocephalus*[83]	M_1_ I_1_ I_2_ M_2_ P_4_ P_3_ C M_3_
*Papio hamadryas hamadryas*[85]	M_1_ M_2_ I_1_ P_3_ P_4_ I_2_ C M_3_
*Theropithecus gelada*[86]	M_1_ I_1_ I_2_ M_2_ P_3_ P_4_ C M_3_
Lophocebus albigena* [82]	M_1_ I_1_ I_2_ [P_3_ P_4_] M_2_ C M_3_
*Mandrillus sphinx*[83]	M_1_ I_1_ I_2_ M_2_ P_4_ P_3_ C M_3_
*Kuseracolobus aramisi*[87]	M_1_ I_1_ I_2_ M_2_ M_3_
*Mesopithecus pentelicus*[87]	M_1_ I_1_ M_2_ I_2_ PP C M_3_
*Piliocolobus badius*[87]	M_1_ [I_1_ I_2_ M_2_] [P_4_ P_3_] [C M_3_]
*Procolobus verus*[87]	M_1_ I_1_ M_2_ I_2_ P_3_ P_4_ C M_3_
*Colobus angolensis*[83]	M_1_ M_2_ I_1_ I_2_ P_4_ P_3_ M_3_ C
*Colobus guereza*[83]	M_1_ I_1_ I_2_ M_2_ P_4_ P_3_ M_3_ C
*Presbytis* sp. [83]	M_1_ M_2_ I_1_ I_2_ M_3_ P_4_ P_3_ C
*Semnopithecus priam*[85]	M_1_ I_1_ I_2_ P_4_ C P_3_ M_2_ M_3_
*Trachypithecus* sp. [88]	M_1_ I_1_ [I_2_ M_2_] P_4_ P_3_ [C M_3_]
*Nasalis larvatus*[87,89]	M_1_ I_1_ I_2_ M_2_ [PP C] M_3_
*Pygathrix* sp. [88,89]	M_1_ M_2_ I_1_ I_2_ M_3_ P_4_ P_3_
*Victoriapithecus macinnesi*[87,90]	M_1_ M_2_ PP
*Hylobates lar*[85]	M_1_ I_1_ I_2_ M_2_ P_3_ P_4_ M_3_ C
*Symphalangus syndactylus*[85]	M_1_ I_2_ I_1_ M_2_ P_4_ P_3_ C M_3_
*Pongo* sp. [41]	M_1_ I_1_ I_2_ M_2_ P_4_ P_3_ C M_3_
*Gorilla* sp. [41]	M_1_ I_1_ I_2_ M_2_ P_4_ P_3_ C M_3_
*Pan troglodytes*[65]	M_1_ I_1_ I_2_ M_2_ [P_3_ P_4_] M_3_ C
*Australopithecus africanus*[91]	M_1_ I_1_ I_2_ M_2_ P_3_ P_4_ C M_3_
*Homo sapiens* (Australian aboriginal) [65]	[M_1_ I_1_] I_2_ C P_3_ [M_2_ P_4_] M_3_
*Homo sapiens* (White American) [65]	[I_1_ M_1_] I_2_ [C P_3_ P_4_ M_2_] M_3_

^a^I_2_ to P_3_ are not erupted.

**Table 2.** Description of the characters used in the ancestral state reconstruction analysis. Characters are treated as unweighted and unordered.

no.	character	states
1	eruption of replacement teeth	0: after molar eruption; 1: first erupted replacement tooth erupts before the last erupted molar
2	premolar eruption sequence	0: 2-3-4; 1: 2-4-3; 2: 4-2-3; 3: 4-3-2; 4: absence of P_2_
3	premolar eruption sequence (if no P_2_)	0: 3-4; 0: 4-3
4	eruption of P_2_ relative to M_3_	0: P_2_ erupts after M_3_; 1: P_2_ erupts before M_3_
5	eruption of P_3_ relative to M_3_	0: P_3_ erupts after M_3_; 1: P_3_ erupts before M_3_
6	eruption of I_1_ relative to the earliest premolar	0: I_1_ erupts after the earliest premolar; 1: I_1_ erupts before the earliest premolar
7	simultaneous eruption of I_1_, I_2_, and C (or I_1_ and I_2_ only)	0: not simultaneous; 1: simultaneous
8	number of incisors erupting after M_2_	0: 3; 1: 2; 2: 1; 3: 0
9	number of premolars erupting after M_3_	0: 3; 1: 2; 2: 1; 3: 0
10	eruption of the incisors relative to M_3_	0: all incisors erupt after M_3_; 1: M_3_ erupts between two incisors; 2: all incisors erupt before M_3_
11	eruption of the incisors relative to the premolars	0: the earliest incisor erupts after the latest premolar; 1: intermediate situation; 2: the latest incisor erupts before the earliest premolar
12	eruption of the premolars relative to M*_2_	0: all premolars erupt after M_2_; 1: at least one premolar erupts before M_2_. (*) Coded as inapplicable if P_1_ is present
13	eruption of M_1_	0: first tooth to erupt; 1: not the first tooth to erupt
14	eruption of P_4_ relative to M_3_	0: P_4_ erupts after M_3_; 1: P_4_ erupts before M_3_

**Figure 2.**
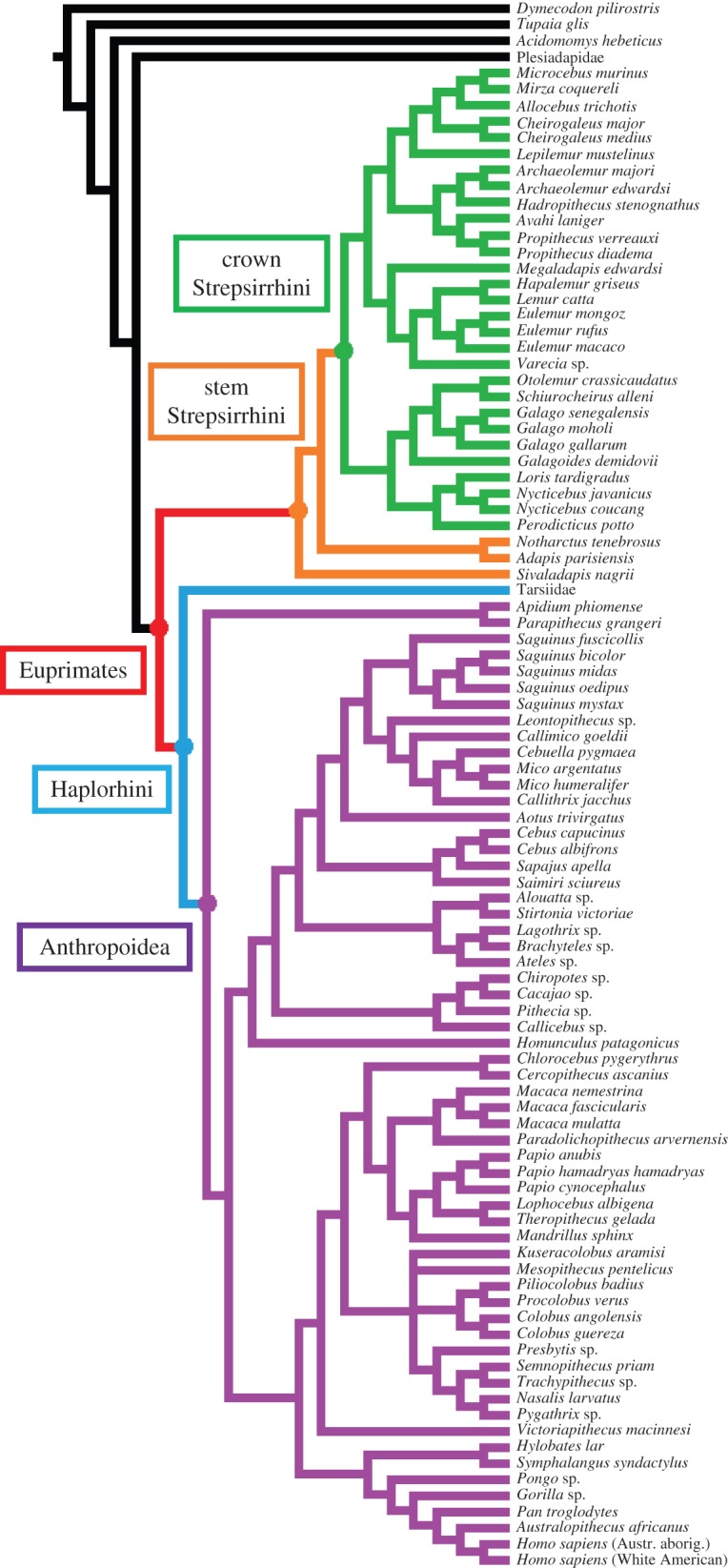
Phylogenetic relationships of the 97 fossil and extant taxa used in this analysis. The ancestral nodes for Euprimates, stem Strepsirrhini, crown Strepsirrhini, Haplorhini and Anthropoidea are indicated. Combined cladogram from Marivaux *et al*. [92], Gunnell [93], Arnold *et al*. [94], Silcox *et al*. [95], Steiper & Seiffert [27], Kay [96], Kistler *et al.* [97] and Seiffert *et al*. [29].

The cladogram (figure 2) used in this analysis is a supertree based on Marivaux *et al*. (used for placing *Sivaladapis* [92]), Gunnell (for *Notharctus* [93]), Arnold *et al*. (for all living primates [94; v. 3], Silcox *et al*. (for plesiadapiforms and *Tupaia* [95]), Steiper & Seiffert (for *Victoriapithecus* [27]), Kay (for *Stirtonia* and *Homunculus* [96]), Kistler *et al*. (for *Hadropithecus* and *Megaladapis* [97]) and Seiffert *et al*. (for *Adapis*, *Apidium* and *Parapithecus* [29]) (see also electronic supplementary material, text S1). Although the trees that we used are considered widely accepted, we acknowledge that there are other phylogenies that might contradict some of the positions in the tree [28,98]. The supertree also reflects discussions on phylogenetic relationships from Strasser & Delson (on *Paradolichopithecus* and *Mesopithecus* [99]), Shoshani *et al*. (on *Archaeolemur* [100]) and Frost (on *Kuseracolobus* [101]). Dental eruption sequences exist for two other platyrrhine species that are not included in the analysis, *Saguinus nigricollis* [102] and *S. graellsi* [46], but Arnold *et al*.'s [94] tree did not include them. Because all *Saguinus* species have very similar dental eruption sequences, we are confident that the exclusion of these two species would not alter the reconstruction of the ancestral anthropoid condition. The outgroups used for the ancestral euprimate node were plesiadapiform taxa. Plesiadapiforms were a group of small, arboreal, archaic mammals widespread during the Palaeocene and the Eocene throughout North America, Europe and Asia. Representatives of plesiadapiforms in our tree include plesiadapids and paromomyids. Here, plesiadapiforms are considered stem primates (following [103–114]). A representative of Scandentia, *Tupaia glis*, as outgroup for Primates (including plesiadapiformes) has been used. Although ideally we would have also included a dermopteran taxon, no permanent dental eruption sequences for dermopterans have been published. Although the lack of dermopterans in the cladogram would have been problematic if the ancestral primate node was reconstructed, the lowest node reconstructed is the ancestral euprimate node. Because plesiadapiforms are inferred to be closer to Euprimates than any other groups (following Silcox *et al*. [95]), under the Outgroup Algorithm [115] they have the greatest impact on polarizing the euprimate node, so it is very unlikely for dermopterans to produce a new unequivocal resolution at that node. Also, the most comprehensive relevant study supports Sundatheria (the combined clade of Scandentia and Dermoptera) as the living sister group of Primates [51], rather than Dermoptera alone [116] or Scandentia alone [117]. This makes Dermoptera no more relevant than the included scandentian in reconstructing the basal euprimate node. The dental eruption sequence of *Dymecodon pilirostris* (Soricomorpha) has been added as a primitive representation of a mammal dental eruption sequence to further constrain the basal euprimate node. Temporal branch length information for living taxa was taken from Arnold *et al*. [94]. The branch lengths for fossil taxa originate from many sources and the choices of dates of appearance of lineages are reported and explained in electronic supplementary material, table S4, and electronic supplementary material, figure S2.

Ancestral state reconstructions were executed in the Mesquite v. 3.01 software package [118], using parsimony. The generalized parsimony algorithm can be applied to optimization of a character of unknown polarity onto a rooted tree, and no additional algorithmic complications are presented by trees containing polytomies [55]. Mesquite also allows missing data when using the parsimony algorithm, but cannot do likelihood calculations with gaps or soft polytomies. Because the data include a significant proportion of relevant fossil taxa, which sometimes produce partially complete eruption sequences, it is important to apply software and an algorithm that can accommodate these limitations, making parsimony the best option. Ancestral permanent dental eruption sequences were reconstructed for five hypothetical ancestors: (i) Euprimates, (ii) stem Strepsirrhini, (iii) crown Strepsirrhini, (iv) Haplorhini, and (v) Anthropoidea. For the ancestral state reconstruction, only lower permanent dental eruption sequences were used, because they are more often reported in the literature, thus increasing the number of taxa available for analysis. Canines were not included in the reconstruction because their time of eruption appears to be influenced by sexual dimorphism [42,82]. Although some primitive primates retain P_1_, eruption data on first premolars are not included in this analysis because the loss of this tooth early in primate evolution renders this character ambiguous at several nodes. To generate the ancestral eruption sequences, all hypothetical ancestors were assumed to have a P_2_. The ambiguities in ancestral state reconstructions for the characters ‘Eruption of P_2_ relative to M_3_’, ‘Eruption of I_1_ relative to the earliest premolar’ and ‘Eruption of incisors relative to premolars’ are resolved with restrictions implied by other characters. For the five nodes studied here, the ancestral state reconstructions result in one permanent dental eruption sequence for each hypothetical ancestor. The ancestral reconstruction analysis was also run without including fossil data (see electronic supplementary material, figure S1 and text S2), to evaluate the effect of fossils on the reconstruction.

As discussed in detail below, several lemurids that were similar to *Darwinius* in body mass and life-history variables were chosen as sources of comparative data. Age-specific body mass data for *Eulemur macaco*, *Eulemur rufus* and *Varecia variegata* (*V. v. variegata*) were taken from the Duke Lemur Center (DLC) database [119]. Although ‘Ida’ has been inferred to be female [17], because this inference is based on negative evidence (see above), we have followed the conservative course of including both male and female data in our analysis. None of the living species are known to be sexually dimorphic, and use of just the female data led to extremely similar results (not shown). The percentage of adult body mass achieved at ‘Ida's’ age at death was determined using the age-specific mass estimated from the Lowess regressions from the three lemurid species, and adult mean body mass estimates from each regression. Lowess regressions were obtained using PAST [120]. Dental eruption times for the three lemurids are taken from Smith *et al*. [42].

It is important to note that some of the specimens from which the data were collected in Eaglen [73], and subsequently reported in Smith *et al*. [42] are currently assigned to other taxa. What Eaglen [73] classifies as *Eulemur fulvus* is currently ascribed to *E. rufus* according to the DLC, and consequently will be referred to as *E. rufus* in this paper. Also, the *V. variegata* sample in Eaglen [73] is composed of a mixture of individuals of *V. variegata* and hybrids of *V. variegata* and *V. rubra* [119]. Therefore, in this paper we are assigning the dental eruption sequence for these specimens to the genus *Varecia* in general.

The dental eruption sequence that we use for *Saimiri* (M_1_ M_2_ I_1_ I_2_ M_3_ P_4_ P_2_ P_3_) is the same one as that used by Franzen *et al*. [17], as the purpose of this paper is to reassess the viability of the original anthropoid model. However, another eruption sequence is known for *Saimiri* [79], which differs in the relative time of eruption of the third molar (M_1_ M_2_ I_1_ I_2_ P_4_ P_2_ P_3_ M_3_). While we use Franzen *et al*.'s [16] eruption sequence throughout the paper, we discuss in the conclusion how Henderson's [79] eruption sequence would influence conclusions about the *Saimiri* model.

## 3. Results and discussion

### 3.1 Ancestral reconstruction of permanent dental eruption sequences

Ancestral dental eruption sequences were reconstructed for five nodes (euprimates, stem strepsirrhines, crown strepsirrhines, haplorhines and anthropoids; table 3), based on the ancestral state reconstruction for the 14 characters (see electronic supplementary material, table S2).

**Table 3.** Reconstructed ancestral permanent dental eruption sequences for five primate nodes (see electronic supplementary material, table S2 for the nodal reconstructions on which these sequences were based). Parentheses () group teeth that in a fossil are either all emerged or all have not emerged yet.

ancestral node	ancestral permanent dental eruption sequence
Euprimates	M_1_ I_1_ I_2_ M_2_ P_2_ M_3_ P_4_ P_3_
stem Strepsirrhini	M_1_ I_1_ I_2_ M_2_ P_2_ M_3_ P_4_ P_3_
crown Strepsirrhini	M_1_ I_1_-I_2_-C M_2_ P_2_ M_3_ P_4_ P_3_
Haplorhini	M_1_ I_1_ I_2_ M_2_ P_2_ M_3_ P_4_ P_3_
Anthropoidea	M_1_ I_1_ I_2_ M_2_ P_2_ P_4_ P_3_ M_3_
*Darwinius masillae*	(M_1_ M_2_ I_1_ P_2_) (I_2_ C M_3_ P_4_ P_3_)^a^

^a^I_2_ to P_3_ are not erupted.

The earliest members of Adapoidea and Omomyoidea are very similar in dental morphology [49], so unsurprisingly there is little variation in eruption sequence inferred for the ancestral nodes. Our ancestral reconstruction suggests two clear trends in the evolution of eruption sequences. The strepsirrhine line is characterized by a primitive dental eruption sequence at the base of stem Strepsirrhini that matches the one inferred for the basal euprimate. Subsequently, this primitive sequence is modified in crown strepsirrhines by the simultaneous eruption of the incisors, along with the canine, in association with the evolution of the toothcomb. The haplorhine line is similarly marked by a primitive basal eruption sequence that resembles the basal euprimate sequence, and then it is characterized by a late eruption of M_3_ at the base of anthropoids. There are several genera of anthropoids in which M_3_ erupts comparatively early (*Saimiri*, *Aotus*, *Pithecia*, *Pygathrix* and *Presbytis* [65,70,79,83,88,89]), but based on the distribution of this trait on this tree this is inferred here to represent evolutionary events occurring in the context of Anthropoidea, like the loss of M_3_ in callitrichines. In contrast to the inferred primitive state for anthropoids, *Darwinius* exhibits early eruption of M_3_ suggesting that it was more strepsirrhine-like. These results therefore make it difficult to determine the relationship of adapoids to either stem strepsirrhines or basal haplorhines, because they both present the same dental eruption sequence. However, these results are less consistent with the Adapoid–Anthropoid hypothesis because adapoids appear to lack the delay of M_3_ eruption, a synapomorphic characteristic of primitive anthropoids.

The crown strepsirrhines show another distinctive feature: the presence of a toothcomb. The eruption of a toothcomb results in the almost simultaneous emergence of the incisors and the canine. The eruption of the toothcomb is generally early for crown strepsirrhines [42,68,69,71,73,74], with the exception of *Archaeolemur edwardsi* [69]. However, the pattern of eruption for the toothcomb does not differ markedly from the eruption pattern of incisors for euprimates, stem strepsirrhines or *Darwinius*, all of which share an early and contiguous eruption of incisors.

The ancestral reconstruction analysis not including fossil taxa provides similar results (electronic supplementary material, table S3), but there is a substantial difference in the final reconstruction. In the analysis that excludes fossils it is not possible to unequivocally reconstruct the primitive premolar eruption sequence for Anthropoidea, with four different states being inferred to be equally parsimonious. This is particularly problematic because the time of eruption of P_2_ is crucial for the study of life history of *Darwinius* specifically. Therefore, the inclusion of fossil data in the analysis is required to resolve relevant ancestral state reconstructions.

### 3.2 Reassessment of the *Saimiri* model

Based on the contrasts between anthropoids and *Darwinius* in the ancestral state reconstruction of dental eruption patterns, *Saimiri* may not be a good model for the growth of *Darwinius*, as previously proposed by Franzen *et al*. [17]. But, contrary to the general anthropoid trend, *Saimiri* is a fast-growing platyrrhine that, like *Darwinius*, exibits an early eruption of M_3_ [70]. The ancestral state reconstruction analysis, however, indicates that this early eruption of the third molar appears secondarily in *Saimiri*. Because the hypothesized relationships of *Darwinius* are to stem anthropoids [30], not Platyrrhini generally, or *Saimiri* specifically, this similarity would necessarily be a case of homoplasy. Also, cebids generally show a late eruption of P_2_ [70,79], with the exception of *Cebus albifrons* [79], in which it appears to be quite variable [72]. *Saimiri* especially stands out among the Cebidae for having one of the latest eruptions of the second premolar. Therefore, this pattern contrasts markedly with that observed in *Darwinius*, in which this tooth is already erupted, with five teeth still remaining unerupted. This has profound implications for calculating the age at death, because it is after the eruption of P_2_ when the sequence is interrupted by death in *Darwinius* [17,44]. It is worth noting that Henderson [79] provides another dental eruption sequence for *Saimiri* in which the relative time of eruption of the M_3_ is markedly later. One of the most convincing arguments in favour of the *Saimiri* model is that squirrel monkeys have, according to the sequence used in Franzen *et al*. [17], one of the most strepsirrhine-like dental eruption sequences among anthropoids, precisely because of an earlier relative eruption of M_3_. In the light of this fact, if Henderson's [79] sequence is correct, *Saimiri* would make an even less appropriate model.

By contrast, both stem strepsirrhines and basal haplorhines would make good models for the growth of *Darwinius*, because of their primitive-looking dental sequences. The only non-anthropoid haplorhine taxa in our sample are tarsiids, which exhibit a dental eruption sequence (table 1) which differs from that inferred for the basal haplorrhine (i.e. extremely early eruption of P_2_, lack of I_2_, and simultaneous eruption of M_3_ and C), making tarsiers a poor choice as model taxa. On the other hand, stem strepsirrhines and *Darwinius* also share early eruption of M_3_ and P_2_. Given that stem strepsirrhines are known only from extinct taxa, without direct information available about their age-specific growth and development, a new growth model based on living strepsirrhines is needed for *Darwinius*.

Three families of strepsirrhines primitively share a dental eruption sequence similar to that of *Darwinius*: Lemuridae, Galagidae and Cheirogaleidae [42,68,73,74]. Galagidae and Cheirogaleidae are significantly smaller than caenopithecids [3]. Generally, in mammals, most life-history variables are correlated to body mass [121], making these very small primates inappropriate models for *Darwinius*. On the other hand, lemurids exhibit similar body masses to caenopithecids [3], which makes them a more reasonable model. Dental eruption sequences are known from six lemurids: *Lemur catta*, *Hapalemur griseus*, *E. rufus*, *E. macaco*, *E. mongoz* and *Varecia* sp. [73]. However, *L. catta* and *H. griseus* would make poor models for the growth of *Darwinius* because the eruption of P_2_ in these two species occurs much later in the sequence, and, as discussed above, this tooth is of critical importance in determining the age at death of this particular specimen. *Lemur catta* and *H. griseus* also possess a premolar eruption sequence of 4-3-2, which is derived in the context of Lemuridae, instead of the primitive 2-4-3 pattern found in the rest of lemurids and stem strepsirrhines. Among the three *Eulemur* species, *E. mongoz* differs the most from *Darwinius* in having a late eruption of P_2_. It would be preferable to apply a model based on species with earlier P_2_ eruptions. Like *E. rufus* and *E. macaco*, *Varecia* has a 2-4-3 premolar eruption pattern and an early P_2_ eruption. Additionally, these fast-growing primates are fairly well studied, making them the best living models available for the growth of *Darwinius*.

The age of eruption of P_2_ in *E. rufus* is 1.14 years, 1.05 years in *E. macaco* and 1.06 years in *Varecia*. Therefore, the age at death of *Darwinius* based on this model would have been between 1.05 and 1.14 years, older than previously suggested (9–10 *months*=0.75–0.83 years [17]).

**Figure 3.**
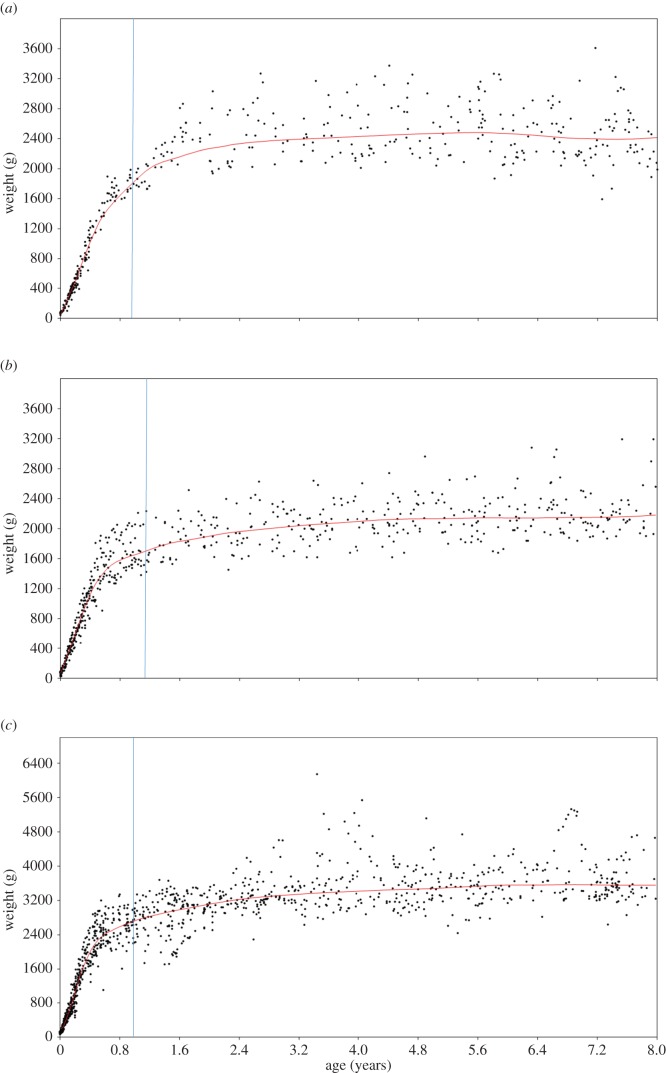
Lowess regressions illustrating patterns of ontogeny for individuals of three lemurid species from birth to the age of 8 years. Smoothing factor of 0.1 for all regressions. (*a*) *Eulemur* *macaco*; (*b*) *Eulemur rufus*; and (*c*) *Varecia variegata*. Vertical lines indicate the supposed interruptions of the sequence in *Darwinius*.

For estimating body mass at death, we used ages at death of 1.05 years for *E. macaco*, 1.14 years for *E. rufus* and 1.06 years for *Varecia*. Individuals of *E. macaco* that aged similarly to *Darwinius* weigh 75.6% of the adult body mass (approx. 1876 g/2481 g; figure 3*a*), whereas individuals of *E. rufus* weigh 78% of the adult body mass (approx. 1699 g/2177 g; figure 3*b*). Finally, *V. variegata* achieves 77.7% of the adult body mass at the relevant age (approx. 2763 g/3556 g; figure 3*c*). Therefore, our lemurid model suggests that ‘Ida’ would have been between the narrow range of 75.6% and 78% of her adult body mass when she died. Franzen *et al*. [17] suggested an estimated body weight at death of 485 g. According to the new lemurid model, the projected adult body mass would be between 622 and 642 g. This is consistent with the adult body mass estimations for other caenopithecids (between 500 and 3500 g [3]), and falls entirely below the estimated adult mass by Franzen *et al*. [17] (650 and 900 g).

## 4. Conclusion

Our ancestral state reconstruction infers the same dental eruption sequences for basal Euprimates, stem Strepsirrhini and basal Haplorhini. These hypothesized primitive sequences resemble that of *Darwinius* in the early eruption of M_3_ and the non-simultaneous eruption of I_1_-I_2_-C in contrast to anthropoids and crown strepsirrhines, respectively. The late eruption of M_3_ in anthropoids and the fact that M_3_ seems to be the next tooth to erupt in *Darwinius* at the moment of her death suggests that anthropoids likely do not provide the most appropriate model for estimating growth in adapoids, including *Darwinius*. The eruption of P_2_ is important for defining the interruption of the sequence in this particular specimen, and the late eruption of P_2_ in *Saimiri* suggests that this genus in particular does not represent a good model for *Darwinius*.

Our results also suggest that eruption sequences carry useful phylogenetic information. Although variable to some extent, higher level primate taxa (e.g. crown Strepsirrhini, Anthropoidea) can be grouped based on different trends in eruption sequences. Therefore, the study of eruption sequences can contribute to our understanding of primate phylogenetic relationships, in a way that allows for the incorporation of fossil material. In this case, the contrast between the inferred late eruption of M_3_ in the common ancestor of Anthropoidea, and the advanced stage of development of this tooth in *Darwinius,* could be interpreted as conflicting with the Adapoid–Anthropoid hypothesis.

The lemurid model for the development of *Darwinius* proposed in this study does not categorically invalidate the *Saimiri* model. However, it is an alternative in closer agreement with the more similar dental eruption sequences found in strepsirrhines. Also, it agrees with the currently most widely supported hypothesis for adapoid relationships: the Adapoid–Strepsirrhine hypothesis. This model suggests an older age at death (1.05–1.14 years, depending on the model used) than previously proposed (0.75–0.83 years [17]). Our model also suggests a narrower range for the projected adult weight (622–642 g) and entirely below the previously proposed (650–900 g [16]), consistent with caenopithecid range of body masses. Although the current data on lemurid growth are sufficient for certain species of lemurids, better documentation of data on growth, and development, and eruption sequences for more lemurid species would certainly improve the quality of potential new models.

## Supplementary Material

Figure S1: Phylogenetic relationships of the 79 extant taxa used in this analysis. The ancestral nodes for Euprimates, Strepsirrhini, Haplorhini, and Anthropoidea are indicated.

## Supplementary Material

Figure S2: Phylogenetic relationships of the 97 taxa (extant and extinct) used in this analysis. Branch numbers correspond to Table S4, which provides the reference for each branch length.

## Supplementary Material

Supplementary tables and text: - Table S1. Character matrix used for the ancestral state reconstruction analysis. - Table S2. Ancestral state reconstruction for five ancestral nodes (Euprimates, stem Strepsirrhini, crown Strepsirrhini, Haplorhini, and Anthropoidea) and 14 characters, including fossil data. - Table S3. Ancestral state reconstruction for four ancestral nodes (Euprimates, Strepsirrhini, Haplorhini, and Anthropoidea) and 14 characters, not including fossil data. - Table S4. Data on branch lengths for fossils and taxa. Branch numbers are from Figure S2. - Text S1. Newick timetree including fossil data. One unit equals 1 million years. - Text S2. Newick timetree not including fossil data. One unit equals 1 million years.

## Supplementary Material

DLC data.xlsx

## Footnotes

1 PMO: Geological Museum, Natural History Museum, University of Oslo; WDC-MG: Wyoming Dinosaur Center, Messel Grube collection.

## Data accessibility

All supporting data are available as the electronic supplementary material.
