# Supplementary figures and images for: Life history of the most complete fossil primate skeleton: exploring growth models for Darwinius
Source: R Soc Open Sci. 2015 Sep 9;2(9):150340. doi: 10.1098/rsos.150340 (PMC4593690; doi:10.1098/rsos.150340)

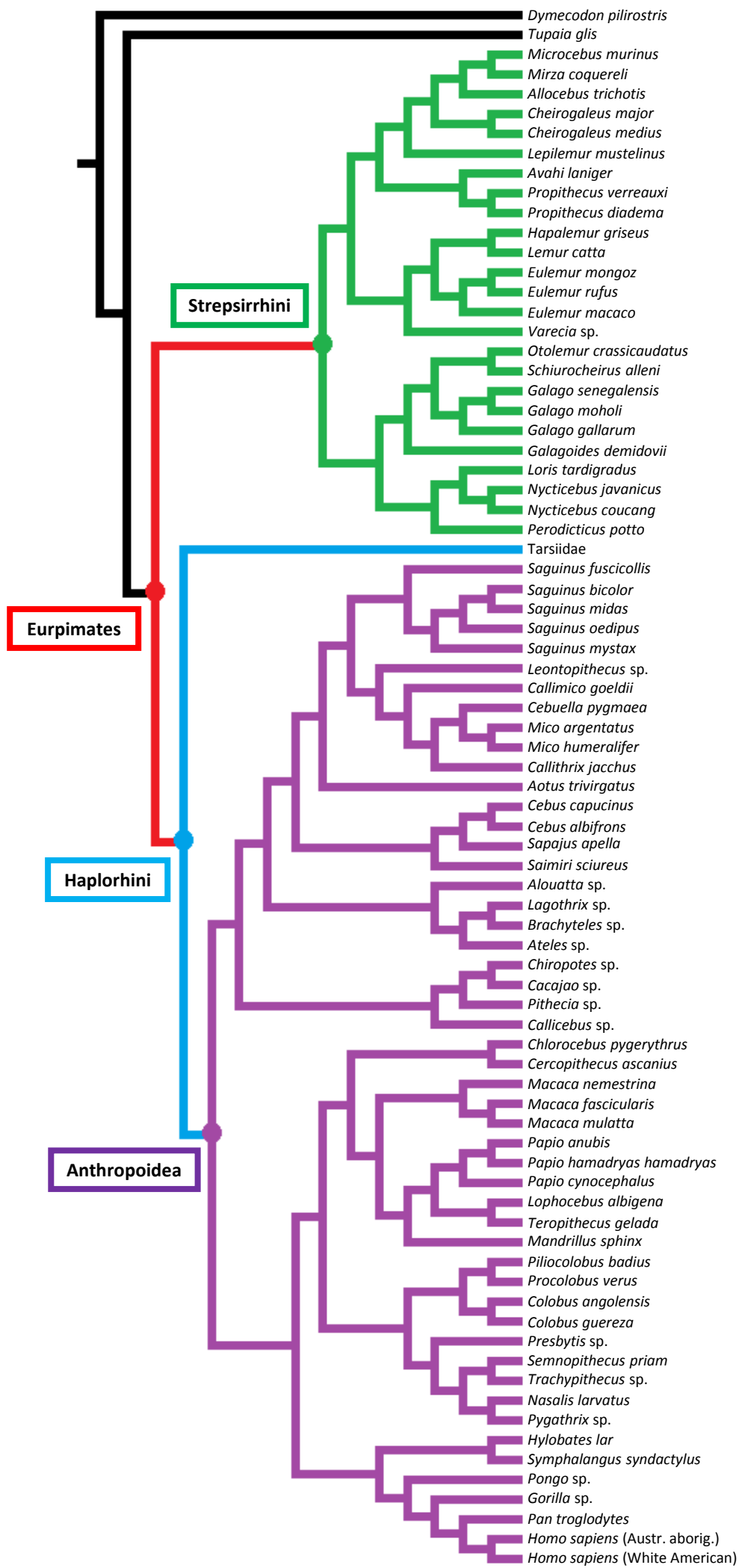

Supplement: Figure S1: Phylogenetic relationships of the 79 extant taxa used in this analysis. The ancestral nodes for Euprimates, Strepsirrhini, Haplorhini, and Anthropoidea are indicated. [file rsos150340supp1.pdf]

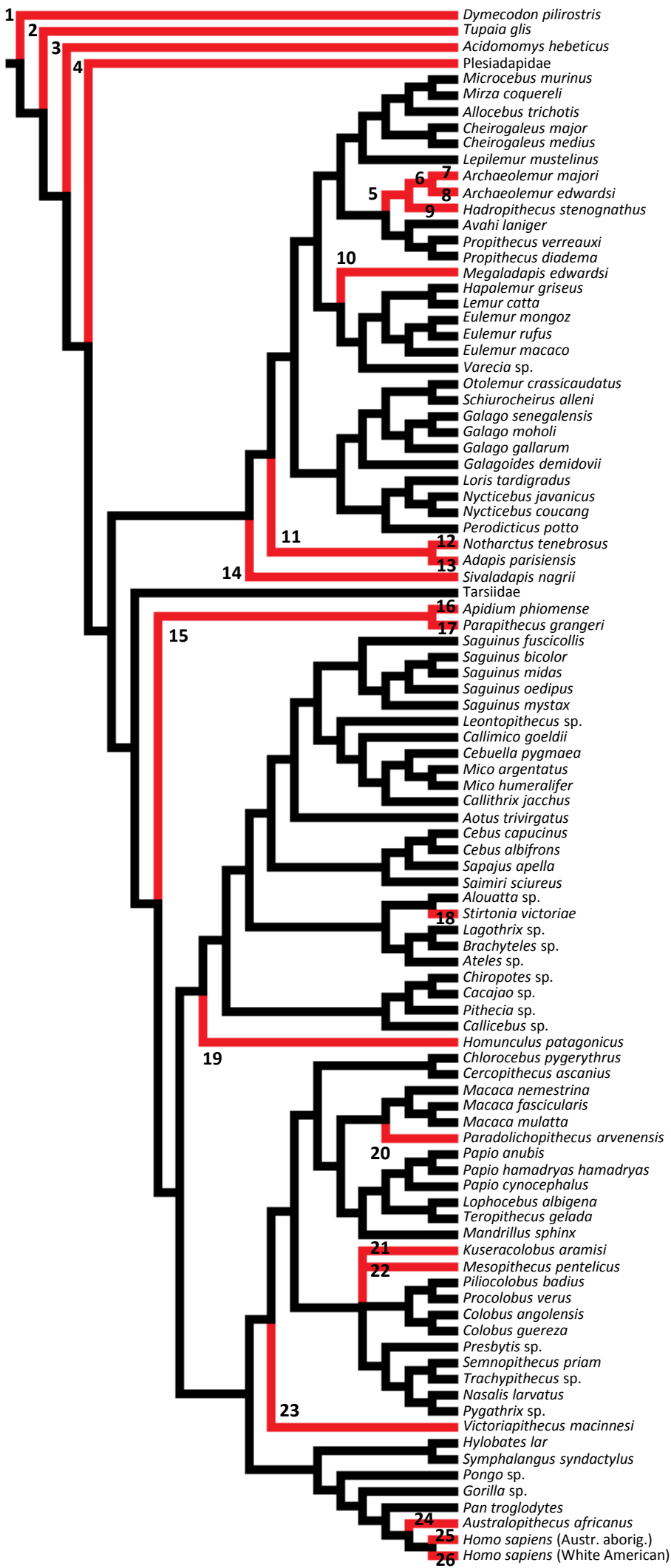

Supplement: Figure S2: Phylogenetic relationships of the 97 taxa (extant and extinct) used in this analysis. Branch numbers correspond to Table S4, which provides the reference for each branch length. [file rsos150340supp2.pdf]
