# Supplementary material for: Life history of the most complete fossil primate skeleton: exploring growth models for Darwinius
Source: R Soc Open Sci. 2015 Sep 9;2(9):150340. doi: 10.1098/rsos.150340 (PMC4593690; doi:10.1098/rsos.150340)
Supplement: Supplementary tables and text: - Table S1. Character matrix used for the ancestral state reconstruction analysis. - Table S2. Ancestral state reconstruction for five ancestral nodes (Euprimates, stem Strepsirrhini, crown Strepsirrhini, Haplorhini, and Anthropoidea) and 14 characters, including fossi [file rsos150340supp3.docx]

**Table S1.** Character matrix used for the ancestral state reconstruction analysis.

|  | **1** | **2** | **3** | **4** | **5** | **6** | **7** | **8** | **9** | **10** | **11** | **12** | **13** | **14** |
| --- | --- | --- | --- | --- | --- | --- | --- | --- | --- | --- | --- | --- | --- | --- |
| *Dymecodon pilirostris* | 0 | 0 | - | 0 | 0 | 0 | 0 | 2 | 0 | 0 | 1 | - | 0 | 0 |
| *Tupaia glis* | 0 | 1 | - | 0 | 0 | 0 | 0 | 0 | 0 | 0 | 1 | 0 | 0 | 0 |
| *Acidomomys hebeticus* | 1 | 4 | 0 | - | 1 | 0 | 0 | 1 | 2 | 1 | 1 | 0 | 0 | 0 |
| Plesiadapidae | 1 | 0 | - | 1 | 0 | - | 0 | - | 1 | - | - | 0 | 0 | 0 |
| *Microcebus murinus* | 1 | 1 | - | 1 | 0 | 1 | 1 | 1 | 1 | 2 | 2 | 0 | 0 | 0 |
| *Mirza coquereli* | 1 | 1 | - | 1 | 0 | 1 | 1 | 3 | 1 | 2 | 2 | 0 | 0 | 0 |
| *Cheirogaleus major* | 1 | 1 | - | 1 | 0 | 1 | 1 | 3 | 1 | 2 | 2 | 0 | 0 | 0 |
| *Cheirogaleus medius* | 1 | 1 | - | 1 | 0 | 1 | 1 | 3 | 1 | 2 | 2 | 0 | 0 | 0 |
| *Allocebus trichotis* | 1 | 1 | - | 1 | 0 | 1 | 1 | 3 | 1 | 2 | 2 | 0 | 0 | 0 |
| *Megaladapis edwardsi* | 1 | - | - | - | - | 1 | 1 | 3 | 1 | 2 | 2 | 0 | 0 | 0 |
| *Lepilemur mustelinus* | 1 | 3 | - | 0 | 0 | 1 | 1 | 1 | 1 | 2 | 2 | 0 | 0 | 1 |
| *Archaeolemur majori* | 1 | 3 | - | 0 | 0 | 1 | 1 | 1 | 0 | 2 | 2 | 0 | 0 | 0 |
| *Archaeolemur edwardsi* | 1 | - | - | - | - | 0 | 0 | 1 | 2 | 0 | 1 | 0 | 0 | - |
| *Hadropithecus stenognathus* | 1 | 3 | - | 0 | 0 | 1 | 1 | 1 | 0 | 2 | 2 | 0 | 0 | 0 |
| *Avahi laniger* | 1 | 4 | 1 | - | 1 | 1 | 1 | 3 | 3 | 2 | 2 | 1 | 0 | 1 |
| *Propithecus verreauxi* | 1 | 4 | 1 | - | 0 | 1 | 1 | 3 | 2 | 2 | 2 | 0 | 0 | 1 |
| *Propithecus diadema* | 1 | 4 | 1 | - | 1 | 1 | 1 | 3 | 3 | 2 | 2 | 0 | 0 | 1 |
| *Hapalemur griseus* | 1 | 3 | - | 0 | 0 | 1 | 1 | 3 | 1 | 2 | 2 | 0 | 0 | 1 |
| *Lemur catta* | 1 | 3 | - | 1 | 1 | 1 | 1 | 1 | 3 | 2 | 2 | 0 | 0 | 1 |
| *Eulemur mongoz* | 1 | 1 | - | 0 | 0 | 1 | 1 | 3 | 0 | 2 | 2 | 0 | 0 | 0 |
| *Eulemur rufus* | 1 | 1 | - | 1 | 0 | 1 | 1 | 3 | 1 | 2 | 2 | 0 | 0 | 0 |
| *Eulemur macaco* | 1 | 1 | - | 1 | 0 | 1 | 1 | 3 | 1 | 2 | 2 | 0 | 0 | 0 |
| *Varecia* sp. | 1 | 1 | - | 1 | 0 | 1 | 1 | 3 | 1 | 2 | 2 | 0 | 0 | 0 |
| *Otolemur crassicaudatus* | 1 | 1 | - | 0 | 0 | 1 | 1 | 3 | 0 | 2 | 2 | 0 | 0 | 0 |
| *Sciurocheirus alleni* | 1 | 1 | - | 1 | 0 | 1 | 1 | 3 | 1 | 2 | 2 | 0 | 0 | 0 |
| *Galago senegalensis* | 1 | 1 | - | 1 | 0 | 1 | 1 | 3 | 1 | 2 | 2 | 0 | 0 | 0 |
| *Galago gallarum* | 1 | 1 | - | 0 | 0 | 1 | 1 | 3 | 0 | 2 | 2 | 0 | 0 | 0 |
| *Galago moholi* | 1 | 1 | - | 1 | 0 | 1 | 1 | 3 | 1 | 2 | 2 | 1 | 0 | 0 |
| *Galagoides demidovii* | 1 | 1 | - | 0 | 0 | 1 | 1 | 1 | 0 | 2 | 2 | 0 | 0 | 0 |
| *Loris tardigradus* | 1 | 1 | - | 1 | 0 | 1 | 1 | 3 | 1 | 2 | 2 | 0 | 1 | 0 |
| *Nycticebus javanicus* | 1 | 1 | - | 1 | 0 | 1 | 1 | 3 | 1 | 2 | 2 | 0 | 1 | 0 |
| *Nycticebus coucang* | 1 | 1 | - | 1 | 0 | 1 | 1 | 3 | 1 | 2 | 2 | 1 | 1 | 0 |
| *Perodicticus potto* | 1 | 1 | - | 1 | 0 | 1 | 1 | 3 | 1 | 2 | 2 | 1 | 1 | 0 |
| *Notharctus tenebrosus* | 1 | 1 | - | 0 | 0 | - | 0 | - | 0 | - | - | - | 0 | 0 |
| *Adapis parisiensis* | 1 | 3 | - | 0 | 0 | - | 0 | - | 0 | - | - | - | 0 | 0 |
| *Sivaladapis nagrii* | 1 | 1 | - | - | - | 1 | 0 | - | - | - | 2 | - | 0 | - |
| Tarsiidae | 1 | 1 | - | 1 | 0 | 0 | 0 | 3 | 1 | 2 | 1 | 1 | 0 | 0 |
| *Homunculus patagonicus* | 1 | 1 | - | 1 | 1 | 1 | 0 | 3 | 3 | 2 | 2 | 0 | 0 | 1 |
| *Saguinus fuscicollis* | - | 2 | - | - | - | 1 | 0 | 3 | - | - | 2 | 0 | 0 | - |
| *Saguinus oedipus* | - | 2 | - | - | - | 1 | 0 | 3 | - | - | 2 | 0 | 0 | - |
| *Saguinus midas* | - | 2 | - | - | - | 1 | 0 | 3 | - | - | 2 | 0 | 0 | - |
| *Saguinus mystax* | - | 2 | - | - | - | 1 | 0 | 3 | - | - | 2 | 0 | 0 | - |
| *Saguinus bicolor* | - | 2 | - | - | - | 1 | 0 | 3 | - | - | 2 | 0 | 0 | - |
| *Leontopithecus* sp. | - | 0 | - | - | - | 1 | 0 | 3 | - | - | 2 | 0 | 0 | - |
| *Callimico goeldi* | - | 2 | - | 1 | 1 | 1 | 0 | 1 | 3 | 2 | 2 | 0 | 0 | 1 |
| *Cebuella pygmaea* | - | 2 | - | - | - | 1 | 0 | 1 | - | - | 2 | 0 | 0 | - |
| *Callithrix jacchus* | - | 3 | - | - | - | 1 | 0 | 1 | - | - | 2 | 0 | 0 | - |
| *Mico argentatus* | - | 3 | - | - | - | 1 | 0 | 1 | - | - | 2 | 0 | 0 | - |
| *Mico humeralifer* | - | 1 | - | - | - | 1 | 0 | 1 | - | - | 2 | 0 | 0 | - |
| *Aotus trivirgatus* | 1 | 3 | - | 0 | 0 | 1 | 0 | 1 | 0 | 1 | 2 | 0 | 0 | 0 |
| *Cebus capucinus* | 1 | 3 | - | 1 | 1 | 1 | 0 | 3 | 3 | 2 | 2 | 0 | 0 | 1 |
| *Cebus albifrons* | 1 | 0 | - | 1 | 1 | 1 | 0 | 3 | 3 | 2 | 2 | 0 | 0 | 1 |
| *Sapajus apella* | 1 | 2 | - | 1 | 1 | 1 | 0 | 3 | 3 | 2 | 2 | 0 | 0 | 1 |
| *Saimiri sciureus* | 1 | 2 | - | 0 | 0 | 1 | 0 | 1 | 0 | 2 | 2 | 0 | 0 | 0 |
| *Alouatta* sp. | 1 | 0 | - | 1 | 1 | 1 | 0 | 3 | 3 | 2 | 2 | 0 | 0 | 1 |
| *Stirtonia victoriae* | 1 | 1 | - | 1 | 1 | 1 | 0 | 3 | 3 | 2 | 2 | 0 | 0 | 1 |
| *Lagothrix* sp. | 1 | 0 | - | 1 | 1 | 1 | 0 | 3 | 3 | 2 | 2 | 0 | 0 | 1 |
| *Ateles* sp. | 1 | 1 | - | 1 | 1 | 1 | 0 | 3 | 3 | 2 | 2 | 1 | 0 | 1 |
| *Brachyteles* sp. | 1 | 0 | - | 1 | 1 | 1 | 0 | 3 | 3 | 2 | 2 | 0 | 0 | 1 |
| *Chiropotes* sp. | 1 | 0 | - | 1 | 1 | 1 | 0 | 3 | 3 | 2 | 2 | 0 | 0 | 1 |
| *Cacajao* sp. | 1 | 0 | - | 1 | 1 | 1 | 0 | 1 | 3 | 2 | 2 | 0 | 0 | 1 |
| *Pithecia* sp. | 1 | 3 | - | 0 | 0 | 1 | 0 | 3 | 0 | 2 | 2 | 0 | 0 | 0 |
| *Callicebus* sp. | 1 | 2 | - | 1 | 1 | 1 | 0 | 3 | 3 | 2 | 2 | 0 | 0 | 1 |
| *Apidium phiomense* | 1 | 1 | - | 1 | 1 | - | 0 | - | 3 | - | - | 0 | 0 | 1 |
| *Parapithecus grangeri* | 1 | 1 | - | 1 | 1 | - | 0 | - | 3 | - | - | 0 | 0 | 1 |
| *Chlorocebus pygerythrus* | 1 | 4 | 1 | - | 1 | 1 | 0 | 3 | 3 | 2 | 2 | 0 | 0 | 1 |
| *Cercopithecus ascanius* | 1 | 4 | 0 | - | 1 | 1 | 0 | 3 | 3 | 2 | 2 | 0 | 0 | 1 |
| *Macaca nemestrina* | 1 | 4 | 1 | - | 1 | 1 | 0 | 3 | 3 | 2 | 2 | 0 | 0 | 1 |
| *Macaca fascicularis* | 1 | 4 | - | - | 1 | 1 | 0 | 3 | 3 | 2 | 2 | 0 | 0 | 1 |
| *Macaca mulatta* | 1 | 4 | 0 | - | 1 | 1 | 0 | 3 | 3 | 2 | 2 | 0 | 0 | 1 |
| *Paradolichopithecus arvernensis* | 1 | 4 | 0 | - | 1 | 1 | 0 | 3 | 3 | 2 | 2 | 0 | 0 | 1 |
| *Papio anubis* | 1 | 4 | 1 | - | 1 | 1 | 0 | 3 | 3 | 2 | 2 | 0 | 0 | 1 |
| *Papio cynocephalus* | 1 | 4 | 1 | - | 1 | 1 | 0 | 3 | 3 | 2 | 2 | 0 | 0 | 1 |
| *Papio hamadryas hamadryas* | 1 | 4 | 0 | - | 1 | 1 | 0 | 1 | 3 | 2 | 1 | 0 | 0 | 1 |
| *Theropithecus gelada* | 1 | 4 | 0 | - | 1 | 1 | 0 | 3 | 3 | 2 | 2 | 0 | 0 | 1 |
| *Lophocebus albigena* | 1 | 4 | 0 | - | 1 | 1 | 0 | 3 | 3 | 2 | 2 | 1 | 0 | 1 |
| *Mandrillus sphinx* | 1 | 4 | 1 | - | 1 | 1 | 0 | 3 | 3 | 2 | 2 | 0 | 0 | 1 |
| *Kuseracolobus aramisi* | 1 | 4 | - | - | - | 1 | 0 | 3 | 3 | 2 | - | - | 0 | - |
| *Mesopithecus pentelicus* | 1 | 4 | - | - | 1 | 1 | 0 | 2 | 3 | 2 | 2 | 0 | 0 | 1 |
| *Piliocolobus badius* | 1 | 4 | 1 | - | 1 | 1 | 0 | 3 | 3 | 2 | 2 | 0 | 0 | 1 |
| *Procolobus verus* | 1 | 4 | 0 | - | 1 | 1 | 0 | 2 | 3 | 2 | 2 | 0 | 0 | 1 |
| *Colobus angolensis* | 1 | 4 | 1 | - | 1 | 1 | 0 | 1 | 3 | 2 | 2 | 0 | 0 | 1 |
| *Colobus guereza* | 1 | 4 | 1 | - | 1 | 1 | 0 | 3 | 3 | 2 | 2 | 0 | 0 | 1 |
| *Presbytis* sp. | 1 | 4 | 1 | - | 0 | 1 | 0 | 1 | 1 | 2 | 2 | 0 | 0 | 0 |
| *Semnopithecus priam* | 1 | 4 | 1 | - | 1 | 1 | 0 | 3 | 3 | 2 | 2 | 1 | 0 | 1 |
| *Trachypithecus* sp. | 1 | 4 | 1 | - | 1 | 1 | 0 | 3 | 3 | 2 | 2 | 0 | 0 | 1 |
| *Nasalis larvatus* | 1 | 4 | - | - | 1 | 1 | 0 | 3 | 3 | 2 | 2 | 0 | 0 | 1 |
| *Pygathrix* sp. | 1 | 4 | 1 | - | 0 | 1 | 0 | 1 | 1 | 2 | 2 | 0 | 0 | 0 |
| *Victoriapithecus macinnesi* | 1 | 4 | - | - | - | - | 0 | - | - | - | - | 0 | 0 | - |
| *Hylobates lar* | 1 | 4 | 0 | - | 1 | 1 | 0 | 3 | 3 | 2 | 2 | 0 | 0 | 1 |
| *Symphalangus syndactylus* | 1 | 4 | 1 | - | 1 | 1 | 0 | 3 | 3 | 2 | 2 | 0 | 0 | 1 |
| *Pongo* sp. | 1 | 4 | 1 | - | 1 | 1 | 0 | 3 | 3 | 2 | 2 | 0 | 0 | 1 |
| *Pan troglodytes* | 1 | 4 | 0 | - | 1 | 1 | 0 | 3 | 3 | 2 | 2 | 0 | 0 | 1 |
| *Gorilla* sp. | 1 | 4 | 1 | - | 1 | 1 | 0 | 3 | 3 | 2 | 2 | 0 | 0 | 1 |
| *Australopithecus africanus* | 1 | 4 | 0 | - | 1 | 1 | 0 | 3 | 3 | 2 | 2 | 0 | 0 | 1 |
| *Homo sapiens* (Austr. aborig.) | 1 | 4 | 0 | - | 1 | 1 | 0 | 3 | 3 | 2 | 2 | 1 | 0 | 1 |
| *Homo sapiens* (White American) | 1 | 4 | 0 | - | 1 | 1 | 0 | 3 | 3 | 2 | 2 | 1 | 1 | 1 |

**Table S2.** Ancestral state reconstruction for five ancestral nodes (Euprimates, stem Strepsirrhini, crown Strepsirrhini, Haplorhini, and Anthropoidea) and 14 characters, including fossil data.

|  | **1** | **2** | **3** | **4** | **5** | **6** | **7** | **8** | **9** | **10** | **11** | **12** | **13** | **14** |
| --- | --- | --- | --- | --- | --- | --- | --- | --- | --- | --- | --- | --- | --- | --- |
| **Euprimates** | 1 | 1 | 1 | 01 | 0 | 01 | 0 | 3 | 1 | 2 | 12 | 0 | 0 | 0 |
| **Stem Strepsirrhini** | 1 | 1 | 1 | 01 | 0 | 1 | 0 | 3 | 1 | 2 | 2 | 0 | 0 | 0 |
| **Crown strepsirrhini** | 1 | 1 | 1 | 01 | 0 | 1 | 1 | 3 | 1 | 2 | 2 | 0 | 0 | 0 |
| **Haplorhini** | 1 | 1 | 1 | 1 | 0 | 01 | 0 | 3 | 1 | 2 | 12 | 0 | 0 | 0 |
| **Anthropoidea** | 1 | 1 | 1 | 1 | 1 | 01 | 0 | 3 | 3 | 2 | 12 | 0 | 0 | 1 |

**Table S3.** Ancestral state reconstruction for four ancestral nodes (Euprimates, Strepsirrhini, Haplorhini, and Anthropoidea) and 14 characters, not including fossil data.

|  | **1** | **2** | **3** | **4** | **5** | **6** | **7** | **8** | **9** | **10** | **11** | **12** | **13** | **14** |
| --- | --- | --- | --- | --- | --- | --- | --- | --- | --- | --- | --- | --- | --- | --- |
| **Euprimates** | 1 | 1 | 1 | 1 | 0 | 01 | 0 | 3 | 1 | 2 | 12 | 0 | 0 | 0 |
| **Strepsirrhini** | 1 | 1 | 1 | 1 | 0 | 1 | 1 | 3 | 1 | 2 | 2 | 0 | 0 | 0 |
| **Haplorhini** | 1 | 1 | 1 | 1 | 0 | 01 | 0 | 3 | 1 | 2 | 12 | 0 | 0 | 0 |
| **Anthropoidea** | 1 | 0124 | 1 | 1 | 1 | 1 | 0 | 3 | 3 | 2 | 2 | 0 | 0 | 1 |

**Table S4.** Data on branch lengths for fossils and taxa not included in Arnold et al.’s [1] tree. Branch numbers are from Figure S2.

| **Branch** | **Length (Ma)** | **Description** |
| --- | --- | --- |
| 1 | 97 – 0 | Based on the divergence date of Laurasiatheria from Euarchontoglires [2]. |
| 2 | 87.9 – 0 | Based on the divergence date of Sundatheria from Primates [3]. |
| 3 | n.a. | The oldest representative on the lineage of the Paromomyoidea is *Paromomys farrandi* (64.52 Ma; [4]), but it conflicts with the molecular date for the basal euprimate (73.003019 Ma). |
| 4 | n.a. | The oldest representative on the lineage of the Plesiadapoidea is *Pronothodectes matthewi* (63.3 Ma [5]), but it conflicts with the molecular date for the basal euprimate (73.003019 Ma). |
| 5 | 24 – 0.00779 | The divergence between Archaeolemuridae and Indriidae is reported to be at 24 Ma [6]. The oldest representative on the lineage of the Archaeolemuridae is *Archaeolemur edwardsi* (7,790 BP; [7]) |
| 6 | 0 | The oldest representative on the lineage of the Archaeolemuridae is *Archaeolemur edwardsi* (7,790 BP; [7]); therefore a value of 0 for used for this branch. |
| 7, 8, 9 | 0.00779 – 0.0005 | The oldest representative on the lineage of the Archaeolemuridae is *Archaeolemur edwardsi* (7,790 BP; [7]). Most subfossil lemurs are reported to have gone extinct 500 BP [8]. |
| 10 | 27.3 – 0.0005 | The divergence between *Megaladapis* and Lemuridae is reported to be at 27.3 Ma [9]. Most subfossil lemurs are reported to have gone extinct 500 BP [8]. |
| 11 | n.a. | No taxon in the lineage of the *Notharctus*-*Adapis* clade (with exclusion of *Sivaladapis*) is known to be older than the molecular date for the basal crown strepsirrhine (62.736553 Ma). |
| 12 | n.a. | Divergence date of *Notharctus* from *Adapis* not known. |
| 13 | n.a. | Divergence date of *Notharctus* from *Adapis* not known. |
| 14 | n.a. | No taxon in the lineage of *Sivaladapis* is known to be older than the molecular date for the basal crown strepsirrhine (62.736553 Ma). |
| 15 | n.a. | The oldest representative of the parapithecid lineage is *Biretia piveteaui* (39 Ma [10]), but it conflicts with the molecular date for the basal anthropoid (46.811821 Ma). |
| 16 | 33.9 – 28.4 | Date range for *Apidium phiomense* [11] |
| 17 | 33.9 – 28.4 | Date range for *Apidium phiomense* [11] |
| 18 | 13.7 – 12.6 | Date range for *Stirtonia victoriae* [12] |
| 19 | 26 – 16.5 | The divergence between *Homunculus* and crown platyrrhines is thought to be between 26 and 30 Ma [13]. The genus *Homunculus* goes extinct by 16.5 Ma [13]. |
| 20 | 8.7 – 1.806 | The oldest representative on the lineage of the Macacini is a *Macaca* sp. from the Miocene of China (8.7 Ma [14]). *Paradolichopithecus arvenensis* goes extinct by 1.806 Ma [15]. |
| 21 | n.a. | Relationships of *Kuseracolobus anamisi* to Colobinae not resolved. |
| 22 | n.a. | Relationships of *Mesopithecus pentelicus* to Colobinae not resolved. |
| 23 | 25.237 – 12.5 | The oldest representative on the lineage of the Cercopithecoidea is *Nsungwepithecus gunnelli* (25.237 Ma [16]). *Victoriapithecus macinnesi* goes extinct by 12.5 Ma [17]. |
| 24 | 4.2 – 2 | The oldest representative on the lineage of the Australopithecinae is *Australopithecus anamensis* (4.2 Ma [18]). *Australopithecus africanus* goes extinct by 2 Ma [19]. |
| 25, 26 | 0.06 – 0 | The human colonization of Australia occurred approximately 60,000 years ago [20]. |

**References for Table S4:**

1. Arnold C, Matthews LJ, Nunn CL. 2010 The 10kTrees Website: a new online resource for primate phylogeny. *Evol. Anthropol.* **19**, 114-118. (doi:10.1002/evan.20251)
2. Murphy WJ, Pringle TH, Crider TA, Springer MS, Miller W. 2007 Using genomic data to unravel the root of the placental mammal phylogeny. *Genome Research* **17**, 413-421. (doi:10.1101/gr.5918807)
3. Janečka JE, Miller W, Pringle TH, Wiens F, Zitzmann A, Helgen KM, Springer MS, Murphy WJ. 2007 Molecular and genomic data identify the closest living relative of Primates. *Science* **318**, 792-794. (doi:10.1126/science.1147555)
4. Clemens WA, Wilson GP. 2009 Early Torrejonian mammalian local faunas from Northeastern Montana, U.S.A. In *Papers on Geology, Vertebrate Paleontology, and Biostratigraphy in honor of Michael O. Woodburne.* (ed LB Albright III), pp. 111-158. Flagstaff, AZ: Museum of Northern Arizona Bulletin 65.
5. Rose KD. 1981 The Clarkforkian Land-Mammal Age and mammalian fauna composition across the Paleocene-Eocene boundary. *University of Michigan Papers on Paleontology* **26**, 1-196.
6. Springer MS *et al*. 2012 Macroevolutionary dynamics and historical biogeography of primate diversification inferred from a species supermatrix. *PLoS One* **7**, 1-23. (doi:10.1371/journal.pone.0049521)
7. Burney DA, Pigott Burney L, Godfrey LR, Jungers WL, Goodman SM, Wright HT, Jull AJT. 2004 A chronology for late prehistoric Madagascar. *J. Hum. Evol.* **47**, 25-63. (doi:10.1016/j.jhevol.2004.05.005)
8. Tattersall I. 1982 *The primates of Madagascar*. New York, NY: Columbia University Press.
9. Kistler L *et al*. 2015 Comparative and population mitogenomic analyses of Madagascar’s extinct, giant ‘subfossil’ lemurs. *J. Hum. Evol.* **79**, 45-54. ([doi:10.1016/j.jhevol.2014.06.016](http://dx.doi.org/10.1016/j.jhevol.2014.06.016))
10. Jaeger J-J *et al*. 2010 Late middle Eocene epoch of Libya yields earliest known radiation of African anthropoids. *Nature* **467**, 1096-1908. (doi:10.1038/nature09425)
11. Simons EL, Bown TM. 1985 *Afrotarsius chatrathi*, first tarsiiform primate (? Tarsiidae) from Africa. *Nature* **313**, 475-477.
12. Hartwig WC, Meldrum DJ. 2002 Miocene platyrrhines of the northern neotropics. In *The primate fossil record* (ed WC Hartwig), pp. 175-188. Cambridge, UK: Cambridge University Press.
13. Kay, RF. 2014 Biogeography in deep time – What do phylogenetics, geology, and paleoclimate tell us about early platyrrhine evolution? *Mol. Phylogenet. Evol.* **82**, 358-374.
14. Qi G. 1979 Pliocene mammalian fauna of Lufeng, Yunnan. *Vertebrata PalAsiatica* **17**, 14-22.
15. Heintz E, Aguirre E. 1976 Le bois de *Croizetoceros ramosus pueblensis*, Cervidae de la faune villafranchienne de la Puebla de Valverde, Teruel (Espagne). *Estudios Geológicos* **32**, 569-572.
16. Stevens NJ, Seiffert ER, O’Connor PM, Roberts EM, Schmitz MD, Krause C, Gorscak E, Ngasala S, Hieronymus TL, Temu J. 2013 Paleontological evidence for an Oligocene divergence between Old World monkeys and apes. *Nature* **497**, 611-614. (doi:10.1038/nature12161)
17. Miller ER, Benefit BR, McCrossin ML, Plavcan JM, Leakey MG, El-Barkooky AN, Hamdan MA, Abdel Gawad MK, Hassan SM, Simons EL. 2009 Systematics of early and middle Miocene Old World monkeys. *J. Hum. Evol.* **57**, 195-211. (doi:10.1016/j.jhevol.2009.06.006)
18. Leakey MG, Feibel CS, McDougall I, Walker A. 1995 New four-million-year-old hominid species from Kanapoi and Allia Bay, Kenya. *Nature* **376**, 565-571.
19. Herries AIR, Pickering R, Adams JW, Curnoe D, Warr G, Latham AG, Shaw J. 2013 A multi-disciplinary perspective on the age of *Australopithecus* in Southern Africa. In *The Paleobiology of* Australopithecus*, Vertebrate Paleobiology and Paleoanthropology* (eds KE Reed, JG Fleagle, RE Leakey), pp. 21-40. Dordrecht, Netherlands: Springer.
20. Roberts RG, Jones R, Spooner NA, Head MJ, Murray AS, Smith MA. 1994 The human colonization of Australia: optical dates of 53,000 and 60,000 years bracket human arrival at Deaf Adder Gorge, Northern Territory. *Quaternary Geochronology* **13**, 575-583.

**Text S1.** Newick timetree including fossil data. One unit equals 1 million years.

Tree Description: (Dymecodon_pilirostris:97,(Tupaia_glis:87.9,(Acidomomys_hebeticus,(Plesiadapidae,((((((((((Microcebus_murinus:14.271122,Mirza_coquereli:14.271122):2.512318,Allocebus_trichotis:16.783441):5.779948,(Cheirogaleus_major:11.388139,Cheirogaleus_medius:11.388139):11.17525):5.865706,Lepilemur_mustelinus:28.429095):2.51681,(((Archaeolemur_majori:0.00729,Archaeolemur_edwardsi:0.00729):0.0,Hadropithecus_stenognathus:0.00729):23.99221,(Avahi_laniger:16.5026,(Propithecus_verreauxi:6.555554,Propithecus_diadema:6.555554):9.947046):7.4974):5.945905):2.318811,(Megaladapis_edwardsi:27.2995,(((Hapalemur_griseus:9.280518,Lemur_catta:9.280518):5.739079,((Eulemur_mongoz:4.153703,Eulemur_rufus:4.153703):1.041696,Eulemur_macaco:5.195401):9.824196):5.523206,Varecia:20.542802):6.757198):5.964716):29.471836,((((Otolemur_crassicaudatus:8.406302,Sciurocheirus_alleni:8.406302):4.958115,((Galago_senegalensis:1.19471,Galago_moholi:1.19471):1.311936,Galago_gallarum:2.506646):10.857772):3.766768,Galagoides_demidovii:17.131185):20.868815,((Loris_tardigradus:24.057527,(Nycticebus_javanicus:2.9545,Nycticebus_coucang:2.9545):21.103028):10.577729,Perodicticus_potto:34.635256):3.364744):24.736553):0.0,(Notharctus_tenebrosus,Adapis_parisiensis)):0.0,Sivaladapis_nagrii):10.266465,(Tarsiidae:68.840033,((Apidium_phiomense:5.5,Parapithecus_grangeri:5.5),((((((((Saguinus_fuscicollis:11.726141,(((Saguinus_bicolor:4.242081,Saguinus_midas:4.242081):4.119345,Saguinus_oedipus:8.361427):1.302045,Saguinus_mystax:9.663472):2.06267):3.986111,(Leontopithecus:15.030065,(Callimico_goeldi:13.232628,((Cebuella_pygmaea:4.789812,(Mico_argentatus:1.73192,Mico_humeralifer:1.73192):3.057892):2.239318,Callithrix_jacchus:7.02913):6.203497):1.797437):0.682187):3.775269,Aotus_trivirgatus:19.487521):0.605006,(((Cebus_capucinus:2.2299,Cebus_albifrons:2.2299):4.219484,Sapajus_apella:6.449384):12.120521,Saimiri_sciureus:18.569905):1.522623):1.228773,((Alouatta:13.7,Stirtonia_victoriae:1.1):1.06024,((Lagothrix:2.35377,Brachyteles:2.35377):6.793621,Ateles:9.147391):5.612849):6.56106):1.411478,(((Chiropotes:2.274028,Cacajao:2.274028):7.368851,Pithecia:9.642879):9.753209,Callicebus:19.396087):3.336691):3.267222,Homunculus_patagonicus:9.5):20.811821000000002,(((((Chlorocebus_pygerythrus:9.848126,Cercopithecus_ascanius:9.848126):5.027053,(((Macaca_nemestrina:6.877996,(Macaca_fascicularis:5.045978,Macaca_mulatta:5.045978):1.832017):1.82204,Paradolichopithecus_arvernensis:6.894):4.153083,((((Papio_anubis:1.277228,Papio_hamadryas_hamadryas:1.277228):0.781218,Papio_cynocephalus:2.058446):3.710717,(Lophocebus_albigena:4.896861,Theropithecus_gelada:4.896861):0.872302):5.5813,Mandrillus_sphinx:11.350463):1.502062):2.022653):6.535196,(Kuseracolobus_aramisi,Mesopithecus_pentelicus,((Piliocolobus_badius:10.64745,Procolobus_verus:10.64745):1.890002,(Colobus_angolensis:3.71468,Colobus_guereza:3.71468):8.822773):2.897705,(Presbytis:13.308102,((Semnopithecus_priam:3.654042,Trachypithecus:3.654042):0.661635,(Nasalis_larvatus:9.634813,Pygathrix:9.634813):3.149834):0.523455):2.127056):5.975217):3.826625,Victoriapithecus_macinnesi:12.737):4.763,((Hylobates_lar:6.598361,Symphalangus_syndactylus:6.598361):13.007584,(Pongo:15.132455,(Gorilla:8.652233,(Pan_troglodytes:6.175879,(Australopithecus_africanus:2.2,('Homo_sapiens_(Austr._aborig.)':0.06,'Homo_sapiens_(White_American)':0.06):4.14):1.975879):2.476353):6.480222):4.473491):10.394055):16.811821):0.0):22.028211):4.162986):0.0):0.0):14.896981):9.1);

**Text S2.** Newick timetree not including fossil data. One unit equals 1 million years.

(Dymecodon_pilirostris:97,(Tupaia_glis:87.9,((((((((Microcebus_murinus:14.271122,Mirza_coquereli:14.271122):2.512318,Allocebus_trichotis:16.783441):5.779948,(Cheirogaleus_major:11.388139,Cheirogaleus_medius:11.388139):11.17525):5.865706,Lepilemur_mustelinus:28.429095):2.51681,(Avahi_laniger:16.5026,(Propithecus_verreauxi:6.555554,Propithecus_diadema:6.555554):9.947046):14.443305):2.318811,(((Hapalemur_griseus:9.280518,Lemur_catta:9.280518):5.739079,((Eulemur_mongoz:4.153703,Eulemur_rufus:4.153703):1.041696,Eulemur_macaco:5.195401):9.824196):5.523206,Varecia:20.542802):12.721914):29.471836,((((Otolemur_crassicaudatus:8.406302,Sciurocheirus_alleni:8.406302):4.958115,((Galago_senegalensis:1.19471,Galago_moholi:1.19471):1.311936,Galago_gallarum:2.506646):10.857772):3.766768,Galagoides_demidovii:17.131185):20.868815,((Loris_tardigradus:24.057527,(Nycticebus_javanicus:2.9545,Nycticebus_coucang:2.9545):21.103028):10.577729,Perodicticus_potto:34.635256):3.364744):24.736553):10.266465,(Tarsiidae:68.840033,(((((((Saguinus_fuscicollis:11.726141,(((Saguinus_bicolor:4.242081,Saguinus_midas:4.242081):4.119345,Saguinus_oedipus:8.361427):1.302045,Saguinus_mystax:9.663472):2.06267):3.986111,(Leontopithecus:15.030065,(Callimico_goeldi:13.232628,((Cebuella_pygmaea:4.789812,(Mico_argentatus:1.73192,Mico_humeralifer:1.73192):3.057892):2.239318,Callithrix_jacchus:7.02913):6.203497):1.797437):0.682187):3.775269,Aotus_trivirgatus:19.487521):0.605006,(((Cebus_capucinus:2.2299,Cebus_albifrons:2.2299):4.219484,Sapajus_apella:6.449384):12.120521,Saimiri_sciureus:18.569905):1.522623):1.228773,(Alouatta:14.76024,((Lagothrix:2.35377,Brachyteles:2.35377):6.793621,Ateles:9.147391):5.612849):6.56106):1.411478,(((Chiropotes:2.274028,Cacajao:2.274028):7.368851,Pithecia:9.642879):9.753209,Callicebus:19.396087):3.336691):24.079043,((((Chlorocebus_pygerythrus:9.848126,Cercopithecus_ascanius:9.848126):5.027053,((Macaca_nemestrina:6.877996,(Macaca_fascicularis:5.045978,Macaca_mulatta:5.045978):1.832017):5.975123,((((Papio_anubis:1.277228,Papio_hamadryas_hamadryas:1.277228):0.781218,Papio_cynocephalus:2.058446):3.710717,(Lophocebus_albigena:4.896861,Theropithecus_gelada:4.896861):0.872302):5.5813,Mandrillus_sphinx:11.350463):1.502062):2.022653):6.535196,(((Piliocolobus_badius:10.64745,Procolobus_verus:10.64745):1.890002,(Colobus_angolensis:3.71468,Colobus_guereza:3.71468):8.822773):2.897705,(Presbytis:13.308102,((Semnopithecus_priam:3.654042,Trachypithecus:3.654042):0.661635,(Nasalis_larvatus:9.634813,Pygathrix:9.634813):3.149834):0.523455):2.127056):5.975217):8.589625,((Hylobates_lar:6.598361,Symphalangus_syndactylus:6.598361):13.007584,(Pongo:15.132455,(Gorilla:8.652233,(Pan_troglodytes:6.175879,('Homo_sapiens_(Austr._aborig.)':0.06,'Homo_sapiens_(White_American)':0.06):6.115879):2.476353):6.480222):4.473491):10.394055):16.811821):22.028211):4.162986):14.896981):9.1);
